# The Practice of Medicine; or a Treatise on Special Pathology and Therapeutics

**Published:** 1842-06

**Authors:** 


### REVIEWS.

---

Art. III.—*The Practice of Medicine; or a Treatise on Special Pathology and Therapeutics.* By Robley Dunglison, M. D., Professor of the Institutes of Medicine, &c., in Jefferson Medical College, Philadelphia; Lecturer on Clinical Medicine, and attending Physician at the Philadelphia Hospital, &c. &c. In two volumes, pp. 1323. Lea & Blanchard, Philadelphia.

There are few, if any, offices that are rewarded with less kindness and thanks, and few, if any, with more dislike and censure, than that of reviewing—more especially *critical* reviewing. Nor is there any one more certain to be thus rewarded, at least in a higher or lower degree. The reason is plain. If the reviewer be at once both competent and faithful—if he be both able and resolved to do strict justice to the subject of his examination, his conscience, taste, and judgment compel him to find some fault with perhaps every work that issues from the press. And the vanity of letters, the avidity of applause, and the pride of authorship, rarely forgive either censure, exception, or even a neglect to praise.

In truth, if the matter be skilfully scrutinized and correctly judged of, it will be found that there are few tasks which man has to encounter, that are more complicated in character, and more difficult of performance than that of the reviewer. These evils arise, not alone from the matter, the spirit, or the mode of composition of the book he has to examine—nor alone from the united influence of the three. To gain approval, and indeed to escape reproach, the reviewer must, to no small extent, frame his critique in accordance with the tastes, the judgments, and even with the fancies and prejudices of several classes of individuals.

Of these classes, the author of the work is of course a very important personage. A single hair-breadth of deviation, made by the reviewer, from what he deems the *right line* of respectful regard, or of virtual commendation, is sure to produce silent dissatisfaction, or open complaint.

Are there, within reach of the critique, any number of contemporary and rival authors, who have recently written on the same or on similar topics? If so, their choler is sure to be stirred up, and their ire awakened, by whatever may be said, which does not, in some way, redound to their credit, at least, if it does not minister to their vanity and self-esteem, by some oblique or direct insinuation, that the palm of authorship is adjudged to them. Is any remark made that can be tortured by construction into an imputation towards them of a want of certain excellencies which others possess? That is magnified into an act of condemnation hardly tolerable. Is no form of reference made to them at all? That is wrought up into an evidence of neglect, which is still more offensive. For, to the vain and the jealous, the bitterest potion that ingenuity can compound, or mischief administer, is, *to let them alone.*

Still more captious and difficult to be pleased is a class of readers, who had never spent a previous thought on the subject under discussion, and who are actual strangers to both science and letters. And this class is querulous and presuming in proportion to their ignorance. As respects merit in composition they have no real knowledge, and therefore supply their want of it by noisy pretension. Another description of readers, who give the reviewer neither annoyance nor concern, is the enlightened, the tasteful, and the inquiring, who examine, observe, and deliberate, and ultimately deem it most safe and advisable to judge for themselves.

In the midst of this throng of petty perplexities, (and more might yet be cited,) what is the hapless reviewer to do? The answer is obvious. To thread his way in safety through the entire labyrinth of difficulties that beset him, is impossible. He must either, therefore, in absolute despair, abandon all attempts to free himself from the entanglement created by them; or he must cut the knots which he cannot untie ,and resolve to gratify his own taste, conscience, and judgment, by awarding justice to the extent of his ability, and allow each class of readers, whether satisfied or dissatisfied with him, to form such views, and adopt such measures, as may be most agreeable to them. And this is the course which we, on the present occasion, are resolved to pursue.

Toward the attainment of the end he ought to have in view, in the examination of a work on which he means to pass a critical judgment, a reviewer may, as a preliminary measure, do something to prepare himself, by asking and answering the following questions.

1. What were the leading objects of the author in the writing and publication of the work? 2. Are these objects laudable and useful, or the reverse? 3. Does the work contain preparations and means well calculated for their accomplishment?

The work whose title forms the heading of this article, and on a brief examination of which we are about to enter, purports to be a system of the "Practice of Medicine; or a Treatise on Special Pathology and Therapeutics." The true interpretation of this announcement is, that the writer designs to prepare a treatise containing a view of the *whole* "Practice of Medicine," not of any *circumscribed* and *insulated portion* of it.

That this is a title, then, of wide scope and lofty pretension, cannot be denied. Its claim is to empire, or universal authority in medicine, unlimited in extent, except by the limits of medical knowledge, and uncontrolled in power, except by a want of professional means and measures not yet discovered, devised, or reduced to practice. In fact the claim is virtually to a scheme of *medical omniscience*, as respects the past, beginning with the fountain-head, or earliest records of the profession, and reaching without a chasm to the present period. To attempt to write *a system* of medicine (and Professor Dunglison has done no less) is to aim at the pinnacle of medical enterprise. It involves an effort to establish, for the time, a professional autocracy, to continue in force and fashion, until new discoveries and further improvements shall call for a more enlarged and enlightened one. To complete the effort then, is to collect and condense into a single treatise a digest of all that is known of the principles and practice of medicine, at the present period, by the Faculty of the world. An omission of any thing important is so far a failure, and bespeaks in the author either carelessness, neglect, or some sort of incompetency to the task he has undertaken.

The leading object of Dr. Dunglison's "Practice" appears to be to improve the profession of medicine, in four points of view, each of the four being highly useful and praiseworthy. And these points are its elements as a SCIENTIFIC—A LITERARY—and A PRACTICAL CALLING; in each of which we say, certainly it ought to have been, and we doubt not was the purpose of our author to contribute to its advancement. Nor is this all that his enterprise exacted of him. It was also his duty, as the fourth point, to facilitate, as far as possible, the attainment of an accurate and complete knowledge of medicine, by the younger and less educated portion of the American Faculty—more especially by medical students.

It does not, however, comport with our present intention, to scrutinize very strictly, much less severely, all the fitnesses or unfitnesses of the work for the promotion of these several forms of improvement. An effort to that effect, besides involving a toilsome and unpleasant task, would require for its accomplishment much more time than we have now at command, and a more protracted and profound investigation, than we are willing to encounter. Nor is this all. The documents growing out of it would fill up a larger space than, in this Journal, could be conveniently allowed to it, conformably to the size and economy of the work. Our review, therefore, (if so it may be called,) will consist chiefly of a succinct series of general observations, accompanied perhaps, or rather followed, by a more formal and detailed discussion of one or more controverted points of doctrine.

As already intimated, the object of Professor Dunglison, in the volumes before us, has probably been, (assuredly it ought to have been,) to improve medicine as a *scientific*, a *literary*, and a *practical* profession, and to facilitate the acquisition of a comprehensive knowledge of it by the American Faculty, especially the junior portion of them, and by medical students.

As regards the SCIENCE of medicine, the Professor has made no improvement in it. He has not even attempted to improve it. In reference to that point, therefore, he is not perhaps justly amenable to blame. To find fault with a writer because he has neglected or failed in what he neither has promised nor is prepared to do, would be illiberal and unjust. And such precisely is the case with our author. He is under no pledge, except by implication, to improve medicine in even the simplest of its scientific qualities. Nor, may we judge from aught he has previously done, do we consider him in any degree fitted for a task of the kind.

Whatever may be the value and efficiency of the Professor's mind, as a condensing lens, to collect within a narrow sphere the lights emitted from a wider one by the original and inventive minds of others, it possesses, if we misunderstand it not, none of the properties of an *electron per se.* It gives not out from itself any of the flashes or coruscations of genius; nor has its course been marked by any of those happy combinations, discoveries, or revelations, which proclaim its possession of creative originality. Though we ourselves do not pronounce it a *positive parasite,* yet, were any other reviewer to do so, we should deem it incautious in an antagonist to enter the lists with him, in a trial of his truth by the ordeal of battle. Indeed might we, without giving offence, institute a comparison on such a subject, we would say that the Professor's mind in its relation to the *minds* of other men, is inferior to the mistletoe in its bearing towards the plant to which it adheres. Though that parasitical product derives from the spot to which it is attached, the matter that nourishes it, yet, by its own powers, it digests, matures, and moulds that matter into its own organism and identity. With the mind of our author, however, the case is different. While it draws its matter of nutriment from the writings of others, it allows that abstracted from each different and dissimilar source, to retain almost entirely its own peculiar form and character. Hence the productions of the Professor, instead of being uniform, consistent, and tasteful compositions, exhibit a crude mosaic or rather patch-work appearance.

We do not deny to Dr. Dunglison either *readiness, hability, or tact* in the *manufacture* (we mean the *compilation*) of books. We acknowledge, on the contrary, that he possesses each in sufficient abundance. But *there* perhaps his book-compounding attributes end. In her allotment to him of the higher and more valuable quality of *judgment,* nature has been sparing, not to say niggardly. Neither are his selections choice, his groupings skilful, nor his arrangements and classifications either able, instructive, or in any way highly appropriate or beneficial. Every thing from his pen, (perhaps we should say *hand,* which may wield other implements besides his pen) is too much encumbered with the minutiæ of detail, and is too scanty in matter of general import. In his nosological catalogues, these faults are broad and striking.

Notwithstanding these remarks, we are compelled to acknowledge, that our author has shown a redeeming share of sound common sense, in his selection of the mode of book-making he pursues. The reason is plain. As already intimated, it is the only mode that he is qualified to practise. Were he to attempt a more elevated and original one, his failure would be equally signal, certain, and humiliating to him. And as relates to himself, this is one of the decrees of fate, which he has had the discernment and good fortune to discover, and the discretion to acquiesce in, and has therefore been sufficiently successful in his literary enterprises. And had he not thus acquiesced, his fortune as an author would have been, of necessity, the reverse. In referference to this point we shall only add, in corroboration of what has been already alleged, that all the works which our author has hitherto published have been the product of the inferior order of the intellectual faculties—of those which, by observation and perception, receive knowledge, and communicate it by language. Of the fruit and finish of the higher intellectual faculties they are altogether destitute.

In as much, then, as the actual improvement of *medical science* consists in one of the three following forms of action, or in the union of all of them—*the discovery of new matter—new creations produced out of matter already known—or new inferences deduced from one or both, in the capacity of premises;* in as much as this is true, and as Professor Dunglison has fallen far short of such distinguished performances, and even shown himself incapable of them; it follows, as a corollary, that, by the work before us, he has effected no improvement in *scientific medicine.*

On the literary standing of the volumes we are compelled to pass a similar judgment. It is *ordinary* in all parts, except where it is *low.* The style of the work is every where loose, crude, and ungraceful, with frequent outbreaks of pomp and bombast. Without pronouncing it positively obscure and muddy, we are fully justified in saying that it is wanting in that cloudless perspicuity, which, in every kind of composition is the most important quality, and, in elementary treatises in science, is altogether indispensable. Criticism might, *in charity,* deal gently with it, were it the production of a *freshman or a sophomore*; but, coming, as it does, from a grave Professor, in the very meridian of mental life, and who prides himself on being a *British scholar,* (educated, we mean, in England) strict justice should be made to visit it; and, all circumstances considered, that is *condemnation beyond reprieve.* To the simplicity and purity, conciseness and vigor of well prepared composition in the Anglo-Saxon tongue, the style and manner of the work are as utter strangers, as if they were not the product of a native pen. As respects many of the writer's customary forms of expression, though we do not pronounce them actual *foreignisms*, yet have they more of a foreign than of a *native* cast. They are at best but a hybridous product between the language of Great Britain and that of sometimes one and sometimes another of the nations of continental Europe. In most instances, however, the adulteration comes from France. Yet does the writer bear the character of a ripe and high bred English scholar.

Nor have we yet referred to all the forms of *affected* and vitiated phraseology in which the Professor indulges himself. In instances, which occur so frequently that we venture not to enumerate them, he employs words under a meaning wholly unauthorized by standard lexicographers, and by the most exemplary writers and speakers of the English language.

Among these misused words stands "invoke" a term of whose employment he is so infatuatedly enamored, that he monotonously and in open violation of etymology and taste, judgment, custom, and all other correct guides and authorities of speech, introduces it on almost every practicable occasion. And in carrying out this strange predilection he not unfrequently so distorts and misapplies it, as to render his meaning in the employment of it equivocal. Of this the following exemplification may serve our purpose. "Pathological anatomy—with heedless or too enthusiastic observers—has too often been *invoked* to establish the former of these positions."

A dislocation of a word more awkward and striking than this can hardly be imagined. And very many similar ones, we say, occur in the employment of the same word, in the course of the work.

"*Revulsion,*" if we misunderstand him not, the Professor often employs as if synonymous with *revolution,* or entire change, and "*economy*" as if identical in signification with *organism, structure, or system.* Now every one may and ought to know, and every medical *scholar* does know, that, when applied to the human body, "economy" strictly means the *action,* or *mode* of *action,* and the native habitudes of the *living organism,* or of some portion of it, not the organism or structure itself. Indeed to our taste even the habitual employment of the *word organism* is exceptionable and repulsive. True, the term itself is legitimate English, and may be occasionally used without much impropiety. But several writers of the day, and quite *distinguished* among them is our author, give it a preference and use it with a frequency which we deem pedantic, affected, and tasteless. In a great majority of cases the word "*organization*" or "system" should receive over it a decided preference. In truth we regard the present fashionable use of it as an unscholar-like dash of *literary dandyism*. Grave Professors should be above a practice so empty and boyish.

True; Professor Dunglison is not, we say, the only medical writer who thus errs in the employment of these terms. Some others still higher perhaps in public confidence and standing, but certainly not lower, have done the same. So much the worse. The larger, stronger, and more authoritative the erring party, the greater is the evil, and the more loudly does it call for correction or removal. Hence we have deemed it our duty, as faithful reviewers, to make it the subject of the preceding strictures. And we regret that a want of room has prevented us from proving, by extracts from our author's "Practice", the truth of the imperfections we have imputed to its style.

Nor have we yet closed our animadversions on what we deem the unscholarlike and sophomorical characteristics of the Professor's style. His predilection for the constant use of a certain set of popular and new-fangled words and phrases, savors not a little of affectation and the unmanly weakness of following the fashion, without calmly reflecting whether it be wise or unwise, right or wrong. This charge we illustrate by specifying the substitution of the words "*normal*" and "*abnormal*" for *natural* or *regular*, and *unnatural* or *irregular*, "*nutritive* or *vital fluid*" for *blood*; "*hyperæmia*," instead of *inflammation* or *congestion* of blood—and scores of others similarly abused, on the exposition of the whole of which we have not leisure at present to dwell. A few more of them, however, we shall here enumerate.

Our author has been guilty of the gross tautological blunder of using the expression "*morbid derangement*," which is tantamount to *morbid morbidity*, or *deranged derangement*.

Another blunder no less discreditable is the substitution of *agency* for *agent*, as in the following sentence.

"If a healthy person exposes his feet to these *agencies*" ("cold" and "moisture"), &c. Now "cold" and "moisture" are not "*agencies*" but *agents*—the former meaning *sorts* or *kinds of action*—the latter the persons or things *that themselves act*. Under no figure of speech, therefore, or construction of language, can either of these words be correctly substituted for the other. Hence nothing but defective scholarship, or unpardonable neglect, could have produced in our author the blunder here committed.

Again,

"As in the case," says the Professor, "of every other disease, not induced by any mechanical cause, the first impression must be made on the *contractility* or *vital properties* of the part with which a morbific body comes in contact."

This clause is also exceedingly faulty and unscholarlike—if indeed by scholarship, be meant a power or state of preparation to *think*, as well as to *write* and *speak*, with correctness and propriety. The phraseology employed in it is indicative of a striking *inaccuracy* of discrimination and jndgment.

To talk of making an "impression on *contractility*", or any other "*vital property*," is to express an idea (if in reality any idea be expressed by such words), in direct opposition to the principles of physiology, as well as to those of common sense.

A "morbific body" does not, in the production of disease, impress "*a vital property*." As well might our author speak of making an impression on a *shadow* or a *thought*. The thing impressed must be substantive. Through the medium of "contractility" or some other "vital property," the "morbific body" impresses the organized part, produces on it a morbid effect, and in that way changes and deranges also the vital property; and disease is the result. For the organ or part is the *fountain* or *parent-stock*, and the vital property the *issue*. And as is the condition of the former then, so must be that of the latter.

We have said that the Professor's style is at times exceedingly cloudy or even muddy and obscure; and the following sentence proves our assertion.

"It is, indeed, impossible to fix, with any thing like precision, as has been attempted, the exact sequence of the disorder of innervation, circulation, and secretion."

If to this clause any one can attach a single clear, well-defined and determinate idea—one single idea, we mean, more certainly and definitely expressed by the language of it than another—if any one can do this, we shall acknowledge him our superior in *clairvoyance*. We can perceive in the phraseology of the extract nothing but a mass of fog and muddiness.

No matter, we repeat, whether other writers, in common with our author, employ these words or not. Their habitual use bespeaks affectedness, and an effort to appear at once both learned and modish.

Professor Dunglison should hold himself above such empty ostentation. Men of mind and attainment should and do engage in substantial operations with the machinery of facts, leaving to boys and inferiors in general to play at crambo, and other forms of mere verbal amusement.

These strictures on style we shall close by observing, that our author makes books too rapidly to avoid in them the commission of errors and blunders of every description which haste and a want of circumspection can produce. His aim seems to be *quantity* rather than *quality*—to compile and publish very *many* books, rather than very *valuable ones.* He appears ambitious to be pronounced able, not to compose a *thousand lines*, like the satirist's poetaster, but to make a *thousand extracts, "stans uno in pede."*

The rapidity with which the Professor prepares his books is productive of two effects, neither of which is favorable to his authorship. It prevents him from displaying in the style of his works either elegance or taste; and it renders his productions too long. Haste in composition conduces to *superfluity* in words, and *scarcity* of thought. To the condensation of matter and style, much time and labor are indispensable. The truth of this may be illustrated by a well known anecdote of a celebrated sermonizer.

The orator was asked how long a time would be requisite for him to deliver, on a given topic, the best sermon he could prepare by the labor of a week? He answered, *An hour.* How long could you preach on the same subject, after the labor of half a week? Ans. *Two hours.* And how long, if you had no time for preparation at all? Ans. *All day.*

Might we therefore, without giving offence, we would, as friends, advise Professor Dunglison to devote more time to the preparation of his books, and abridge the number of them. By good-naturedly complying he may oblige his readers, by making his productions shorter, and improve his own reputation, as a writer, by making them better.

We strongly suspect that the worthy Professor has allowed himself to be too much flattered by the encomiums passed by certain reviewers, or rather *noticers*, on the prolificness of his mind. He has been highly complimented, on account of having published an unusual number of works, within a given number of years. We beg to assure him that this is a fallacious ground of compliment. True; by hasty book-making he *may* advance his *pecuniary* interest—but never will he, in that way, improve the condition of either his scientific or his literary reputation. We shall only add, that the reviewer who justly censures a writer for his faults, is more truly and beneficently his friend, than the flatterer who applauds him for excellencies which he does not possess. And, without aspiring to the standing of a seer, we venture to predict, that, provided Professor Dunglison receive what we have here said in a proper tone and temper of mind, and make the proper use of it, he will find hereafter more substantial ground to thank us for our animadversions, than he will to thank those who have injudiciously or dishonestly flattered him, for their praises.

That these free and unceremonious strictures on Professor Dunglison's "Practice of Medicine," will be attributed by some persons to unfriendliness of feeling toward him, on our part, is hardly to be doubted. The charge, however, if preferred, will be groundless. We are not unfriendly toward him. But, in the words of Brutus to Cassius, "We do not like his faults." And his style is not only faulty in itself; it is calculated to do mischief by the production of similar faults in others.

With the junior portion of the medical profession in our country, more especially with pupils, the Professor is a popular—I might perhaps say, a *favorite* writer. And favorites are likely to be followed and copied, particularly in the attributes which render them favorites—and in none of these more so, provided they be writers, than in that of style and manner.

It is more than probable, therefore, perhaps even certain, that the style of Professor Dunglison will become a model for imitation by young medical writers, whose taste and judgment are yet immature, and easily vitiated and led astray. And if so, the consequence is plain. Such imitation will operate to the deterioration of the style of the medical writings of our country. Hence, to contribute *our* part toward the prevention of the evil, we have deemed it our duty to pass on the Professor's composition the foregoing strictures. And, should the measure become necessary, or even advisable, we hold ourselves prepared, as already mentioned, to prove their correctness by extracts from the work.

But the most important element of our author's production is yet to be examined. We allude to its practical one. What will be its bearing on the treatment of disease, the alleviation of human suffering, and the preservation of human life? Will it, in these respects, be productive of any change, amounting to a truly valuable improvement? To be able, under the approbation of our conscience, and the sanction of our judgment, to answer this question affirmatively, would be eminently gratifying to us.

But such gratification it does not fall to our lot to experience. In a practical point of view, the volumes before us present to us nothing with which we were not already familiar, when first we opened them. Indeed, neither on the score of principle nor practice have we been able to discover any thing in them positively new. If to this they furnish an exception, it consists in a catalogue of technical formulæ, to which, by enlightened physicians, possessed of candid, liberal, and independent minds, who rely and act on principles and generals, rather than on petty points and particulars, but little if any importance is attached. To speak more plainly and definitely on the subject, we are unable to discover any solid or even specious ground of belief, that a single disease has yet been, or is likely to be hereafter cured, or a single life saved, through the instrumentality of Dunglison's "Practice of Medicine,,' which might not have been as well, and as certainly effected, had the volumes never been issued from the press. All this, however, would have been excusable in our author, had he, without the addition of any thing of novelty or improvement by himself, in either fact or principle, embodied in his work a correct account of the most efficient and successful modes of practice now pursued in a very large proportional part of our own country. This, however, he has failed to do—for what reason we presume not to know. We refer to the *fact alone;* because in that only do we take any concern. In truth, moreover, we doubt not a little whether he has faithfully recorded the most vigorous and successful forms of practice pursued in any of the sickly parts of our country. Of western and southern regions only, however, do we speak with positiveness.

Instead of examining and recommending, or even treating with common respect, what is well known to be the *most* successful, if not the *only* successful mode of treating the endemic and epidemic complaints of the Mississippi Valley, the Professor has somewhat superciliously, and therefore exceptionably scouted and condemned it. On this point we speak with confidence, and tell him plainly, that the form of practice which he dignifies and honors with the epithet "*philosophical*,"

because he deems it the most enlightened, judicious, and efficient, would, if employed in the treatment of the giant diseases of the West and South, prove, in many cases, virtually an abetment of manslaughter. If it did not itself kill, it would allow disease to do the work. The want of judgment of our author on this subject, and his rejection of, or unacquaintance with, the issue of all experience, are matters of astonishment to us. And that astonishment is increased, by the overweening confidence and utter disregard for the sentiments of others, with which he proclaims and urges his opinions, when contrasted with the limitedness of his opportunities for acquiring the requisite amount of correct information. For, in the knowledge of the diseases of hot climates and valley-situations obtained by observation and experience (and no other sort is of any avail) he is wholly unversed. The Professor is not indeed an advocate for the feeble "gum-water" practice of Broussais, an attempt to introduce which into Philadelphia was recently made by a popular teacher. May we judge, however, from many of his receipts, prescriptions, and general directions, his scheme of therapeutics is, in not a few instances, but little superior.

Without pretending to any *positive* information in the case, we feel authorized to believe, that Professor Dunglison has never been to the south of the 37th or 38th degree of north latitude, and that, even when there, the place of his sojourn was *mountainous*—its elevation, as respects *temperature* and its effects on health, being equal to several degrees of northing. That he should possess then any amount of *self-acquired* knowledge of the complaints of warm and sickly climates, is absolutely impossible. Nor will he himself contend for the contrary opinion. And we repeat, without fear of refutation, that, in relation to successful practice in such places, no other kind of medical knowledge is of any efficiency. Knowledge acquired exclusively from books, unaccompanied by experience, is, for practical purposes, unavailable and useless.

For proof of the correctness of this statement, we need only refer to our author's preface. He there reveals to us the extent and variety of his travels and residences. And they are both considerable. According to his showing, he has been in the north of England, and in Edinburgh and Paris, as a student of medicine, in London as a practitioner, and, as a teacher and a practitioner, in the Universities of Virginia and Maryland, and in the Jefferson Medical College, and one of the hospitals in Philadelphia. And there terminates the career of his shiftings of residence, and its several concomitants; having reached from about north latitude 54° or 55° to north latitude 37° or 38°, and over a stretch of longitude much greater. But he has never, we repeat, been a resident of a hot and sickly climate. He has never even visited one of the true southern and western States of the Union, and hence knows, expxrimentally and practically, nothing of the character and treatment of their diseases. And he, like all other men, is incompetent to teach, either orally or in writing, what he does not understand. Yet to this truth, which is as indisputable a maxim as any in mathematics, he seems (may we credit his own words) to be an entire stranger.

In the preface to his "Practice" he first tells us that the "great principles of Pathology and Therapeutics are the same every where." And of the truth of that sentiment no ghost need rise from the dead to convince us; in as much as all men fully admit it without such evidence. But all men as fully and firmly reject his opinion, when he immediately adds, that "one who has been well grounded in those principles can exercise his profession with as much satisfaction to himself, and advantage to the sick, in the scorching presidencies of British India, as in the more temperate regions of our own country."

True; a well educated physician can treat successfully the complaints of those burning regions when he has learnt, by a skilfully directed experimental apprenticeship, to do so. But to contend that he can thus treat them, on his first encounter with them, merely because he is "well grounded in the principles of Pathology and Therapeutics" as they are taught in medical schools—to contend for a notion so empty as this, is to set recklessly at defiance, and stubbornly gainsay the sound dictates of common sense, as well as the verdict of observation and experience. Were the fancy of Professor Dunglison on this topic correct, where would be the value of elaborate treatises written exclusively on the diseases of hot climates—or indeed of any particular climates or localities whatever? Such productions would be supererogatory and useless, and therefore mere encumbrances in our libraries. Let young men "ground themselves well" in the mere "principles of Pathology and Therapeutics," and, in our author's opinion, they become on the instant, efficient practitioners. Hence, the worthlessness of the writings of Lind, Cleghorne, Hillary, Hunter, Moseley, Chesholm, and the entire class of writers on tropical complaints!! They are all superseded and rendered stale and valueless by our author's "Treatise on special Pathology and Therapeutics"!!—because it contains an exposition of the principles of those two branches of medical knowledge! A position more notoriously fallacious than this it is impossible to imagine. Yet is it a fair deduction from the visionary doctrine avowed by our author.

At another of the prefatory passages of the Professor's "Practice of Medicine" we are greatly surprised. It shows, if we mistake not its nature and bearing, a looseness of thought, and a *want* of *comprehension*, or a *miscomprehension* of the correct relation of cause and effect, quite unworthy of its distinguished author. It is as follows.

"Hence the medical officers of our army and navy, especially the latter, whose duties carry them to every part of the globe, are found to be as successful in the management of the cases that fall under their care in distant regions, as they would be in the treatment of those that prevail in the spot where they received their medical education."

To pronounce the statement contained in this extract *a gross exaggeration* would be no doubt deemed language excessively discourteous and exceptionably strong. Yet does the case admit of language still stronger and plainer. The statement involves a *positive misrepresentation.* "The medical officers of our navy" are *not* "as successful in the management of the cases that fall under their care in *distant regions*," as in the treatment of our own domestic diseases, with which they are familiar. The truth of this appears from scenes of distress and desolation that have often occurred in the West Indies, and other intemperate and sickly climates. Our sailors and marines, new to such places, have been attacked, soon after their arrival, by forms of disease equally new to the "medical officers" of the ships. And the issue of the visitation has told a tale exceedingly different from that which our author has inserted in his preface. The mortality among the "new-comers" has been often appalling. Why? Chiefly because the "medical officers" have been unversed in the character and treatment of the prevailing complaint. Yet have they not been altogether uninstructed in the "general principles of Pathology and Therapeutics."

There exist perhaps two principal causes why our sailors and marines are swept off so alarmingly, under the complaints which assail them, soon after their arrival in hot and sickly places. Their constitutions being yet unacclimated renders them more liable to fatal attacks; and their physicians being in a great measure strangers to their maladies (being certainly inexperienced in the treatment of them),are less skilful in the duty devolved on them. But this state of things becomes gradually amended. Time acclimates the constitutions of the officers and crews; and experience gives skill and tact to the physicians and attendants. And, as the natural consequence of such changes, the disease becomes less formidable and fatal.

All things considered, we are at a loss to conceive how a medical writer could broach and attempt to vindicate a more visionary and untenable notion, than that on which we have briefly animadverted. An effort to prove the existence of an "*elixir vitæ*," or to manufacture such an article, would hardly surpass it in wildness and folly. And in the amount of mischief our author's hypothesis would inevitably produce, could a belief in it be widely extended, it would throw far into the back-ground all the dreams in which the alchymists have indulged. But, fortunately for the credit of medicine, and the interest of man, the glaringness of its fallacy, by preventing its adoption, will extinguish in embryo the evils that would arise from it.

Few things in medicine have surprised us more than the inveterate pertinacity, with which Professor Dunglison, and perhaps some other writers and teachers in the Atlantic States, adhere to their notion, that they are themselves actually competent to the exposition and treatment of the herculean diseases of the West and South, *which they have never witnessed*—and (still more erroneous and assuming) that they can communicate to their pupils a thorough practical knowledge of that which they have never themselves had an opportunity deliberately to study, nor even momentarily to inspect!! The Professor, as we are told, is a sturdy disbeliever in *mesmeric clair-voyance*, and even imputes to those, who are holders and advocates of the opposite belief, a condition of mind scarcely short of *imbecility and foolishness.* Yet does he labor, with the zeal of a fanatic and the perseverance of a martyr, to impress on the world an implied admission of his own *omnivisient* clair-voyance—(may we coin an extraordinary name for so extraordinary a power)—a faculty so clear in its function, and so boundless in its sphere of action in him, that he can, by means of it, while seated in his study in Philadelphia, explore successfully the specialties, as well as the general characters of the complaints of the Mississippi Valley, and learn how to cure them!! "*Risum teneatis amici!!*" So easy and natural is it for gentlemen of a certain organization, temperament, and caliber, to strain at gnats, as respects the performances of others, and to swallow camels and moose-deer themselves!!

True; in justice to our author, and the other Atlantic writers and teachers, who claim such super-eminent *clair-voyance*, in relation to western and southern complaints, we are compelled to acknowledge that their claim is in *words*, rather than in acts. Hence, when they are making preparation to write, speak, or lecture on a western disease, they consult either western men, or western writings, or both, for the materials they want. Nor can thay possibly derive them of the slightest value from any other source, except by crossing the mountains, and practising their profession for a time in the West. And were they to adopt that expedient, they would soon be convinced of the truth of our remarks—that they must either abandon, in some most material points, their eastern modes of treating disease, or surrender all hope of successful results.

Such, we venture to assure them, has been the case with all pupils, who, educated exclusively in Atlantic schools, have passed the mountains, and pursued their profession in the Mississippi Valley—with all, at least (and their number has not been small) with whom it has fallen to our lot to converse on the subject. They have been compelled to exchange the practice inculcated on them by eastern teachers, for one exceedingly different, derived from western example, observation, and experience.

In truth, to many eastern physicians whom we could name, a sojourn of a few months in the West would be eminently serviceable. The experiment, if properly made, would teach them not a few things of which they have no knowledge, and which notwithstanding they deeply need.

In point of feeling, it would impart to them a more liberal share of modesty than they now possess. Does any one ask us, *in what way* it would thus act? We answer, by teaching them a more accurate knowledge of themselves, *in comparison with others*—ay; even in comparison with *back-woods* men! It would enable them to know, and compel them to confess that, in all new-settled tracts of country, there is no deficiency in the knowledge and fitness of the inhabitants, as relates to any of the essentially requisite pursuits and duties of life. All things necessary to the varying wants and homely comforts of the people are not only done, but satisfactorily done. And this is perhaps as true of the treatment and cure of diseases, as it is of any other forms of business. Many a practitioner, who travels from house to house through the forest, carrying his powders, pills, and potions in his saddle-bags, could give instructive lessons, and set a profitable example, even to *eastern profesors,* in relation to the treatment of the sick whom he visits. He could convince them that he surpasses *even themselves,* notwithstanding their accumulated and highly and justly-prized stores of *book-knowledge,* as respects the cure of the complaints of the place. Nor to this would Professor Dunglison himself form an exception. In the management of the diseases of the forest, the comparatively unlettered and plain-mannered physician of the "back-settlement" would be superior to the book-learned and polished physician of the metropolis.

Of this superiority the reason is plain. The country practitioner possessed of it is, by long experience, familiar with the diseases he prescribes for, while he of the city, for want of experience, is a stranger to them.

Let it not be imagined, from these remarks, that we, in the slightest degree, undervalue medical literature. Far from it. Our only meaning is, that as regards the cure of disease, it is much less valuable than medical experience. And in this every physician of common sense will heartily concur with us.

Though Professor Dunglison is not alone possessed of the conceit that he is completely accomplished in the knowledge of the nature and treatment of western complaints, and that he can therefore expound them and communicate an accurate acquaintance with them to pupils, as thoroughly and instructively as western teachers; yet, if we mistake him not, he contends for that notion more openly and doggedly, than any other individual in the schools of the east. In truth it has long been our belief, that the Professor has a propensity unusually strong, to prefer a claim to the possession of *unacquired* knowledge—a kind of knowledge, we mean, derived from neither reading, observation, experience, nor oral intercourse, nor indeed from any other source without, or apart from, himself. It would seem to be made up exclusively of *internal, spontaneous,* and *un-labored-for* ideas or fancies, for which he is indebted not even to *himself,* but to a peculiarly bounteous endowment of Nature, which the capricious goddess has bestowed on him, but on nobody else. The following anecdote may serve as a farther illustration of our meaning.

About twenty years ago, (a few years perhaps more or less,) Dr. Dunglison arrived by invitation in the United States, as one of the Professors in the University of Virginia. Soon after that period the writer of this article had the pleasure of an introduction to him, and found him confident in the belief that, though he had scarcely perhaps, at the time of the interview, felt an American pulse, or made a single visit to an American sick room, he had, notwithstanding, a complete knowledge of American diseases, and was perfectly competent to their treatment and cure, and also to become an instructor in them to others.

Nor is this statement made in the form of an inference from a casual conversation. It is the substance of an opinion that was frankly avowed. And although, during the conversation, the Professor himself was perfectly calm, courteous, and self-possessed, one or two of his colleagues deported themselves differently, when the correctness of the belief was delicately questioned. An insinuation quite civilly made, that an English physician *might* be surpassed and even instructed, in his profession, by an American, appeared to be regarded in the light of an indignity. And thus was the conversation suddenly interrupted, or the subject of it changed. Under this head we shall only add, that we have known intimately many European physicians of great respectability, who had migrated from their own country, and become resident practitioners in this. And Professor Dunglison excepted, we are, at this moment, unable to call to mind a single one, who did not frankly confess, that, on his arrival in the New World, he found it *really new* in most things; and that, in particular, he found himself a stranger to the successful treatment of American diseases, until he had become acquainted with it by experience. And, in the nature of things, the acknowledgment could not be other than ingenuous and veritable.

If the foregoing strictures and disquisitions be founded in truth and justice, (and our aim has been conscientiously to that effect,) it necessarily follows that in neither a *scientific*, a *literary*, nor a *practical* point of view, has the profession been improved by the volumes we are examining. Is it then to be hence inferred, that medicine is in no way benefited by the work? We answer, no. A few useful purposes in the profession, the publication creditably subserves. Some of these are the following.

In the first place, it makes a part of the great scheme of *labor-saving* and *power-giving* means and contrivances of the day. It brings together and presents to its readers, under a succinct and condensed form, a vast amount of medical matter, which no physician could collect for himself, without a most tedious and toilsome examination of detached works; and which, from a want of leisure and books, the great body of physicians in the United States could never collect. By its copious references to authorities, it serves to those who are solicitous of further information on given topics, as an index to libraries to which they may have access. Nor is this all.

It presents to the physicians of the United States a very valuable and praiseworthy example of literary research, ambition, and perseverance. And were that example judiciously analyzed and deciphered, improved and followed out, it might be rendered the most profitable attribute of the book. It furnishes a lesson replete with instruction, as to what may be accomplished by labor alone, without the aid of *genius* or *talents.* For, in the preparation of his system of "Practice," the Professor has not made the slightest manifestation of either. He has shown himself to be, in the strict and entire meaning of the phrase, a "*working man,*" possessed of the excellent and essential qualities of assiduty, faithfulness, and perseverance, but, in our view, of little or nothing higher. And the example he has thus put forth ought to prove exceedingly useful and encouraging to all moderately gifted men, who constitute an immense majority of the Caucasian race itself, and the entire mass of all the other races. Even among the Anglo-Saxon and Anglo-American Caucasians, who are in no small degree superior to the other varieties of men, the proportion of the true-highly gifted is extremely small. And even of that number but few employ their talents to the best effect. The reason is plain. To such persons *labor* is irksome and repulsive, and *pleasure* fascinating. They are mostly an intractable compound of opposites and extremes. Hence, trusting to their high natural endowments, for the attainment of their wishes, and the gratification of their ambition, they reject the disagreeable and cling to the pleasing. Hence also they fail of that eminence they might easily attain, and of that usefulness, in whose production they might be greatly instrumental.

By the compilation of his "Practice of Medicine," then, we repeat, Professor Dunglison has done much to gratify and encourage, the great body of moderately gifted men. He has practically shown them, what it is important they should know, that by *industry*, *zeal*, and *perseverance alone,* without the aid of mental powers above *medioc-*

*rity*, much may be done to acquire reputation and reward ambition. For that, in the estimation of a great majority of that portion of the Faculty of our country, who may read his "Practice," his reputation will be enhanced by it, is hardly to be doubted.

In the case before us, therefore, the Professor's example and good fortune will do much in confirmation of the great and important truth, that, where nature, merely by her choice and abundant endowments, confers distinction and its concomitants on *one* man, active energy, enterprise, and perseverance, confer them on thousands. Hence, were this proposition rigidly analyzed, and carried out to its full extent, it would clearly reveal and establish the fact, that men are the makers and marrers of their own fortunes; that they have the control of their own destinies, far beyond what is generally admitted, and that they are therefore to themselves the only FATE to whose influence they are subjected, and whose *fiat* they are positively compelled to obey. So true is this, that those who labor for standing and distinction resolutely and steadily, without faltering or flinching, and under the direction of common sense and sound moral principles, rarely fail in the attainment of their purpose.—One topic more, and our task will be suspended for the present, if not finally closed.

As this article, if read at all, will be read chiefly by the physicians of the West and South, it may probably be expected of us that we should offer an opinion more definite and positive respecting the fitness or unfitness of the volumes before us to subserve, *in particular*, the interests of practical medicine, in the Mississippi Valley.

To this expectation, supposing it to exist, we can reply, *to our own satisfaction*, in a single sentence. Others, of course, notwithstanding all we have said, or can say, will exercise their right to judge for themselves.

After the most strict and impartial examination into the matter and character of the work in question, we are unable to discover in it the shadow of fitness to benefit practical medicine in the Mississippi States. It is a subject of regret to us therefore, that, as honest and conscientious reviewers, we cannot cordially recommend it to western patronage. There yet exists as deep and positive a necessity for an able and efficient work on the nature and treatment of western diseases, as there did before Dunglison's "Practice" was committed to the press; or as there would have done, had the Professor never delivered a lecture nor wielded a pen.

It was our intention to offer a few remarks on one or two points of doctrine maintained by our author in the volumes before us. But the unlooked for length to which this article has already extended, compels us to postpone the completion of our purpose until another occasion.

